# The Preliminary Education of the Dentist

**Published:** 1903-02-15

**Authors:** C. M. Wright


### THE PRELIMINARY EDUCATION OF THE DENTIST.

BY DR. C. M. WRIGHT, D.D.S.

The entire subject of education, its object and methods has undergone radical changes in this country during the past third of a century. Still it is impossible to believe that we have arrived at the goal, or at a solution of the problem of the education of either mind or body. Schools, colleges and universities with the traditions of the past clinging to them are influenced by the times.

Modern business methods seem to be the *sine qua non* of individual and institutional success. The man or school succeeds in proportion to his or its keenness of appreciation of the education of to-day, and his or its activity and ability to grasp and adapt conditions to the new demands.

Notwithstanding scientific and philosophical conservatism and pessimism in educational and business matters, present success depends upon the acceptance of modern facts as the best available tools with which to work.

In the dental school with which I have been associated for twenty years, the entire system of teaching and managing has changed. We could not keep our doors open, if we should return to the ways of the past. There has been a steady lengthening of time necessary to complete the course. From two years of four or five months each, and a single course of lectures, repeated each year, to a graded course of three years of seven or eight months each; and now to a four years' course, with indication of increased time and higher attainments as the next notch. Twenty years ago no special preliminary education was required. A man might then like Cincinnatus, leave his plow in the furrow, and become a full fledged student of a dental college. Many did come from the jeweler's and the carpenter's bench, or the blacksmith's anvil. Now they must come from the high school. In a few years the Bachelor degree will be required of all Medical and dental students in this country. It may be a curious, as well as a significant side fact, that dentistry has made vast strides in science and the art, during these twenty years, and the strides have been made by the "poorly equipped graduate" of the jeweler's and blacksmith's days. Similar stories can be told of Harvard and of Miami, whose distinguished alumni did not have the benefit of modern systems and yet have been leaders in the professional and business worlds.

Now, how can I answer the question propounded to me by your editor?

In the first place, we are compelled to recognize the fact that to-day a definite amount of regular preparatory schooling is required, for entrance to a dental college. Can the student then best fit himself by preliminary work in his college looking toward the Bachelor of Arts, or the Bachelor of Science degree, if he expects to adopt Dentistry as a profession?

This must be a matter of purely individual opinion for the present. One man argues that as dentistry is surgical and mechanical—a manual profession, dealing with living tissue and inorganic matter—with physiology, pathology, surgery and therapeutics on the one hand, and electricity, metals, ceramics, machines and appliances on the other, that the course of preparatory study should be along the lines of biology and physics, and that language and philosophy might be left out. Another believes that education is not intended to fill minds with facts or cases (as the lawyer would call them), but to train the intellect during its early and rapid development, to habits of attention, memory and judgment. This one would have a foundation laid for future culture which will be broad enough to permit the erection of any special structure, whether that be of a scientific, artistic, social, political, business or professional character. He would insist upon attention being given to those studies which tend to open broader views of the past and present. Such studies as language, history, philosophy and logic, or the so-called classical course, would be favored by him.

There are arguments apparently unanswered on both sides of the question. We can offer pros and cons on either side. Now, when I am asked to write my opinion on the subject, if I favor one or the other of these sides, it will be because of some intangle prejudices; or of some unrecorded impression from long experience with students of all grades of preliminary preparation; or of some half digested notions based on questions of physiological waste and repair—foot pounds of energy, etc—or, of an inner consciousness of needs of my own, mixed with desires and hopes, failures and ambitions; together with a lot of other inexpressible emotions that have lurked for years about my waking hours of work and my dreaming hours of rest.

The sum of these fancies would, I believe, influence my vote in favor of a *classical foundation*—this to be laid in a "small college" where a "scholarly atmosphere" surrounds and impregnates a venerable campus; where traditions of simple but noble lives abound; where moral worth stands out in as bold relief as worldly success. I regard the influence such studies in such a place, as of the highest value as a persistent force, at the time of life when college days are fitting the boy for the beginning of any special university course, whether of Law, Medicine, Dentistry, or other arts and sciences, business, so-called, included.

Years ago I watched a graduate of Miami painting the outside of his mother's house. The workman beside him on the ladder remarked, "You paint pretty well for a man who never learned the trade."

"Do you know why?" said the graduate.

"Because I studied Latin under Prof. Bishop. That's why I can beat you at painting a house." And if I wanted to find a good dental student to-day, I think I should look for the boy who has studied Latin under some Prof. Bishop, and Greek under some Prof. Elliot, and with his elementary science and college philosophy, I should consider the young man well equipped to begin at the bottom of Dentistry.

As the courses in Medicine, Dentistry and Law are lengthening, and as these professional schools are trying to train the modern student in practice, and thus endeavoring to save the patient or the client from the inexperience of the beginner, as President Elliot has so ably pointed out in one of his lectures, I should make the academic course shorter, so that impatience and idleness (from possible nerve exhaustion in the over training), might not be as apparent in the special schools. That is, I should try to so arrange the studies of a boy, that he might graduate from a small college at from 18 to 20 years of age with his soul filled with the dignity of his membership in good standing of the "Aristocracy of Learning." He would then bring enthusiasm to the fresh professional course, and at the age of 23 or 24 he would begin his life's work with a stock of useful energy, which would send him ahead with a momentum due to force, and fit him for weathering all storms, away from the protecting umbrella of any Alma Mater.—*Miami Student.*

